# Contemporary critical limb ischemia: Asian multidisciplinary consensus statement on the collaboration between endovascular therapy and wound care

**DOI:** 10.1007/s12928-018-0523-z

**Published:** 2018-04-13

**Authors:** Osami Kawarada, Kan Zen, Koji Hozawa, Shinobu Ayabe, Hsuan-Li Huang, Donghoon Choi, Su Hong Kim, Jiyoun Kim, Taku Kato, Yoshinori Tsubakimoto, Tasuya Nakama, Shigeo Ichihashi, Naoki Fujimura, Akihiro Higashimori, Masahiko Fujihara, Tomoyasu Sato, Bryan Ping-Yen Yan, Skyi Yin-Chun Pang, Chumpol Wongwanit, Yew Pung Leong, Benjamin Chua, Robbie K. George, Yoshiaki Yokoi, Hisashi Motomura, Hideaki Obara

**Affiliations:** 1 Department of Cardiovascular Medicine, National Cerebral and Cardiovascular Center, 5-7-1 Fujishiro-dai, Suita, 565-8565 Japan; 2 Department of Cardiovascular Medicine, Ikuwakai Memorial Hospital, Osaka, Japan; 3 Department of Cardiovascular Medicine, Kyoto Prefectural University of Medicine, Kyoto, Japan; 4 Department of Cardiology, New Tokyo Hospital, Matsudo, Japan; 5 Department of Plastic Surgery, Yao Tokushukai General Hospital, Yao, Japan; 6 Division of Cardiology, Taipei Tzu Chi Hospital, Buddhist Tzu Chi Medical Foundation, New Taipei, Taiwan; 7 Division of Cardiology, Severance Cardiovascular Hospital, Yonsei University College of Medicine, Seoul, South Korea; 8 Department of Cardiology, Busan Veterans Hospital, Busan, South Korea; 9 Department of Orthopedic Surgery, Busan Veterans Hospital, Busan, South Korea; 10 Department of Cardiology, Rakuwakai Otowa Hospital, Kyoto, Japan; 11 Department of Cardiology, Kyoto Second Red Cross Hospital, Kyoto, Japan; 12 Department of Cardiology, Miyazaki Medical Association Hospital, Miyazaki, Japan; 13 Department of Radiology, Nara Medical University, Kashihara, Japan; 14 Division of Vascular Surgery, Tokyo Saiseikai Central Hospital, Tokyo, Japan; 15 Department of Cardiology, Kishiwada Tokushukai Hospital, Kishiwada, Japan; 16 Department of Radiology, Tsuchiya General Hospital, Hiroshima, Japan; 17 Department of Medicine and Therapeutics, Prince of Wales Hospital, The Chinese University of Hong Kong, Sha Tin, Hong Kong; 18 Department of Surgery, Pamela Youde Nethersole Eastern Hospital, Chai Wan, Hong Kong; 19 Department of Vascular Surgery, Siriraj Hospital, Bangkok, Thailand; 20 Department of Vascular Surgery, Cardiac Vascular Sentral Kuala Lumpur, Kuala Lumpur, Malaysia; 21 Department of Vascular Surgery, Vascular and Interventional Centre Singapore, Mount Elizabeth Novena Specialist Centre, The Farrer Park Hospital, Singapore, Singapore; 22 Department of Vascular Surgery, Narayana Hrudayalaya and Mazumdar Shaw Medical Centre, Bengaluru, India; 23 Department of Plastic and Reconstructive Surgery, Osaka City University, Osaka, Japan; 24 Department of Surgery, Keio University School of Medicine, Tokyo, Japan

**Keywords:** Peripheral artery disease, Interdisciplinary, Collaboration, Ischemia, Bacterial infection, Foot deformity

## Abstract

The burden of peripheral artery disease (PAD) and diabetes in Asia is projected to increase. Asia also has the highest incidence and prevalence of end-stage renal disease (ESRD) in the world. Therefore, most Asian patients with PAD might have diabetic PAD or ESRD-related PAD. Given these pandemic conditions, critical limb ischemia (CLI) with diabetes or ESRD, the most advanced and challenging subset of PAD, is an emerging public health issue in Asian countries. Given that diabetic and ESRD-related CLI have complex pathophysiology that involve arterial insufficiency, bacterial infection, neuropathy, and foot deformity, a coordinated approach that involves endovascular therapy and wound care is vital. Recently, there is increasing interaction among cardiologists, vascular surgeons, radiologists, orthopedic surgeons, and plastic surgeons beyond specialty and country boundaries in Asia. This article is intended to share practical Asian multidisciplinary consensus statement on the collaboration between endovascular therapy and wound care for CLI.

## The beginning of collaboration between endovascular and wound care specialists in Asia

Appropriate diagnosis of critical limb ischemia (CLI) has been underappreciated. Although endovascular therapy has evolved around the world during the last decade, the endovascular community in both developed and developing Asian countries has distinct variations in clinical practice frameworks, device availability, regulations, and reimbursement. Consequently, there is a broad spectrum in maturity or dominancy of the endovascular specialty among cardiologists, vascular surgeons, and radiologists by county. In parallel with advancements in endovascular therapy, awareness of the importance of wound management still remains underdeveloped. Thus, dissemination of comprehensive approaches in different disciplines for treatment of CLI is urgently needed.

In 2010, the first meeting of the Endovascular Asia (formerly, Bay Area Endovascular Summit) took place as a grassroots forum for interaction between cardiologists, vascular surgeons, radiologists, orthopedic surgeons, plastic surgeons, vascular nurses, wound care nurses, vascular ultrasonography technologists, and clinical engineering technologists beyond national boundaries to appreciate the need for multidisciplinary practice and harmonize endovascular therapy and wound care. During Endovascular Asia 2017 in Osaka, the first Asian CLI meeting session called CLI Asia was convened on December 2, 2017, to generate Asian multidisciplinary expert consensus on the essential diagnostic and treatment strategy with participation by endovascular and wound specialists from Japan, Taiwan, South Korea, Hong Kong, India, Singapore, Thailand, and Malaysia. This article is intended to share the practical consensus statement based on the collaboration between endovascular therapy and wound care beyond specialty and country borders that reflect discussions at that meeting.

## An emerging framework for critical limb ischemia in Asia

**Fig. 1:**
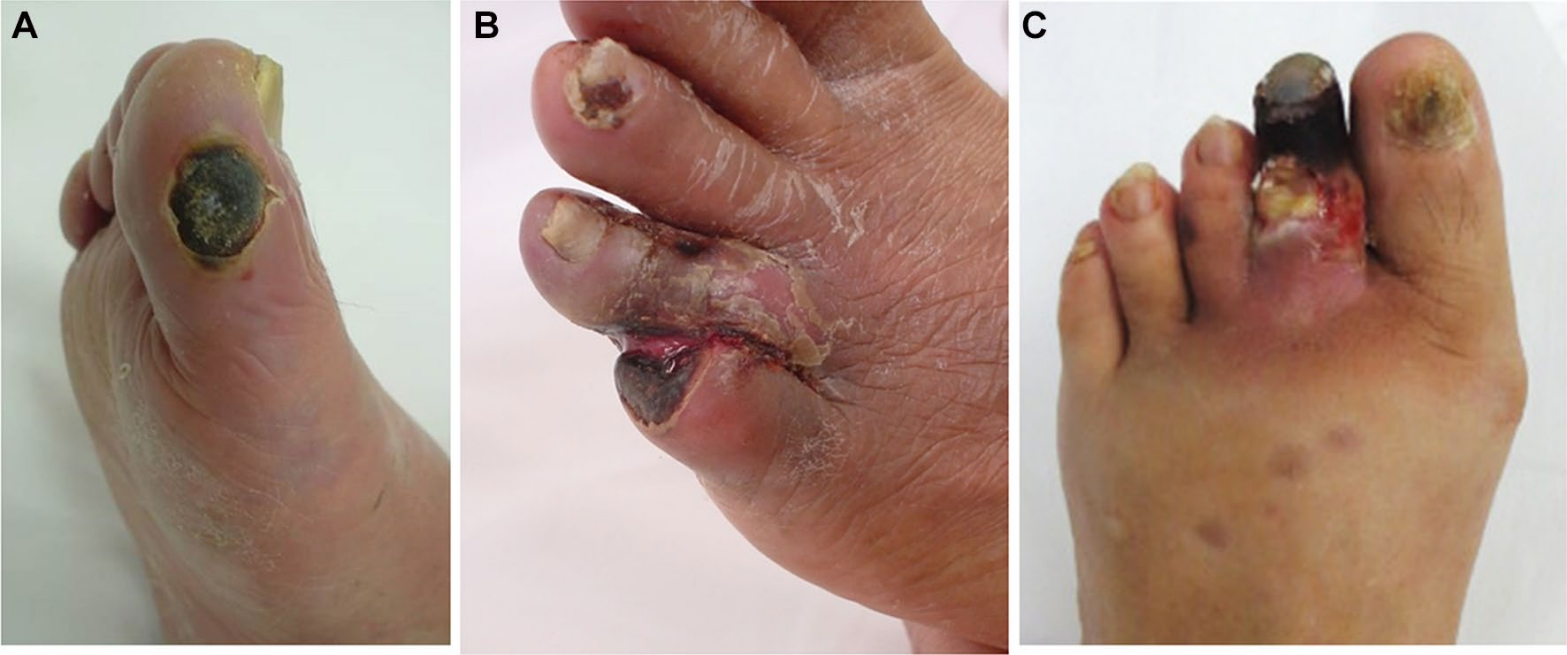
Representative ischemic tissue loss. **a** Traditional CLI. Traditional CLI such as nondiabetic or non ESRD-related CLI can typically cause dry tissue loss. **b** Diabetic CLI or ESRD-related CLI. Most of the diabetic or ESRD-related CLI cause wet tissue loss, suggesting significant bacterial infection. This is the most challenging subset of CLI. **c** Diabetic foot. Although wound appearance is similar to CLI, foot microcirculation is preserved

Asia comprises about 60% of the world population, with intercountry variations in economic status and health-care systems. Currently, there is a projected increase in the burden of peripheral artery disease (PAD) and diabetes in Asia [1, 2]. Asia has the highest incidence and prevalence of end-stage renal disease (ESRD) in the world [3]. Therefore, most Asian patients with PAD may also have diabetes or ESRD. CLI in the context of diabetes or ESRD is the most advanced and challenging subset of PAD (Fig. 1); given these pandemic conditions, this is an emerging public health issue in Asian countries. Diabetic and ESRD-related CLI has complex pathophysiology that includes dysfunction of the macrocirculation due to extensive atherosclerotic lesions, dysfunction of the microcirculation including arteriovenous shunting, bacterial infection, and diabetic foot deformity associated with neuropathy [4]. Diabetes mellitus and ESRD can change the nature of peripheral artery disease, especially infrapopliteal artery disease. Vessel calcification, endothelial dysfunction, subsequent thrombosis, and vasoconstriction can cause disturbances in the macrocirculation and microcirculation [5–7]. Foot ischemia can be further impaired by neuropathy. Arteriovenous shunting due to autonomic neuropathy, exogenous factors such as excessive loading due to motor and sensory neuropathy, and diabetic foot deformity can cause neuroischemic ulcers or gangrene [8]. In addition, hyperglycemia and impaired immunological responses can increase susceptibility to bacterial infections of ischemic wounds. Subsequent infectious arteritis in the small arteries of the foot can exacerbate ischemia. Consequently, polymicrobial foot infections can range from minimal superficial infections to deep infections such as osteomyelitis; these infections can create a physical barrier against re-epithelialization and amplify the risk of sepsis. Therefore, compared to CLI patients without diabetes or ESRD, CLI patients with diabetes or ESRD have approximately twice the risk of wound healing failure, reintervention, and major amputation or death even after endovascular therapy [9, 10]. If CLI patients can meet the following conditions: less cardiovascular comorbidity, functional recovery expectancy after revascularization, good quality autogenous vein conduit, and an estimated life expectancy of 2 years or more, bypass-first strategy might be considered as an initial revascularization strategy [11, 12]. However, since most Asian countries have only a small group of dedicated vascular surgeons compared to Western countries [13], Asian CLI patients might have less chance to undergo bypass surgery. In consideration of Asian situations, endovascular therapy with sophisticated wound management is the mainstream approach for contemporary CLI.

## Assessment of the microcirculation and differential diagnosis

**Fig. 2:**
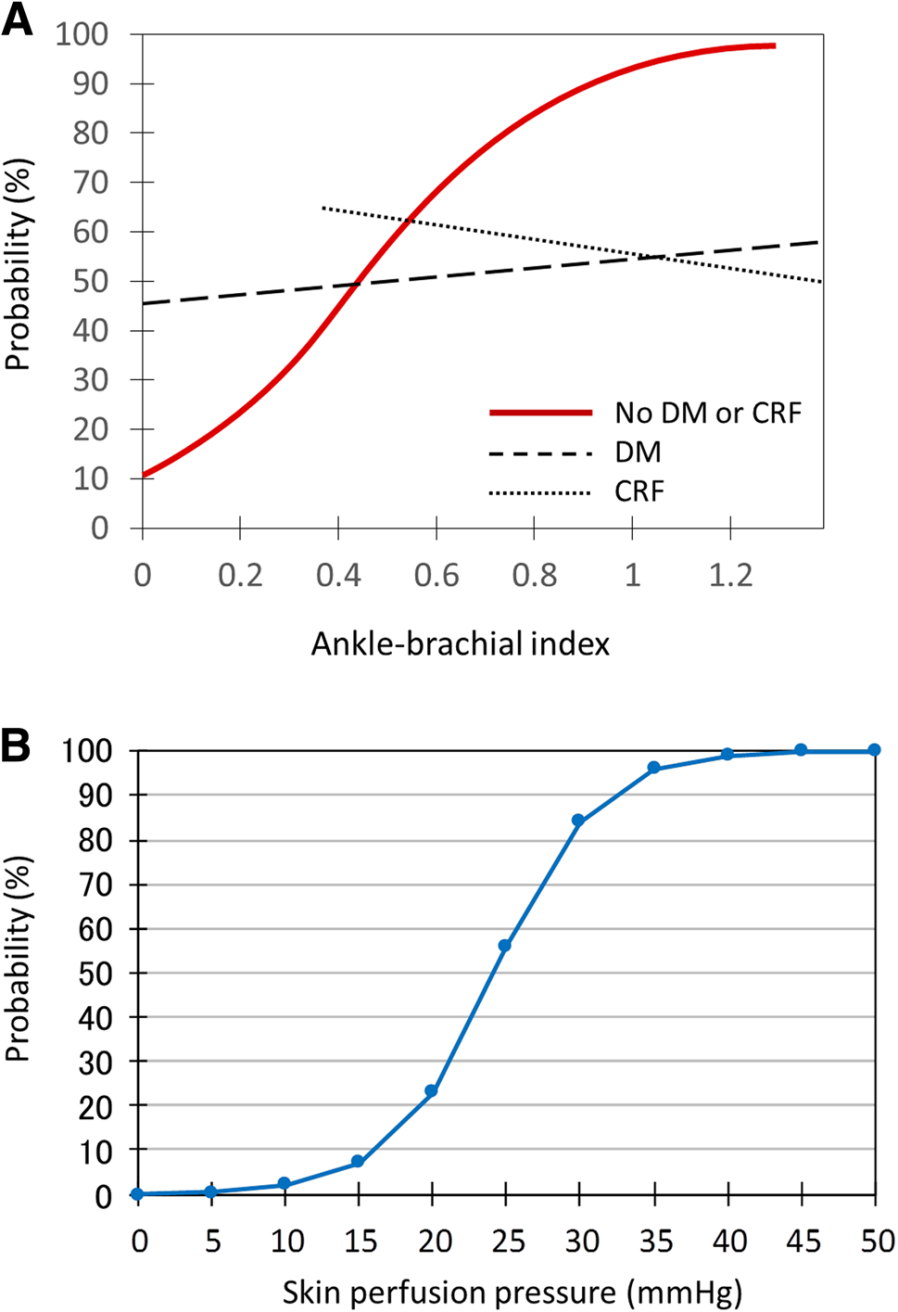
Assessment of the macrocirculation and microcirculation. **a** Relationship between ABI and probability of ischemic wound healing (modified from reference [15]). In patients without diabetes or renal failure, there is a significant relationship between ABI and wound healing. However, in patients with diabetes or renal failure, which commonly co-exist with critical limb ischemia, no relationship between ABI and wound healing is observed, suggesting that assessing the role of the macrocirculation is limited in the setting of critical limb ischemia. *DM* diabetes mellitus, *CRF* chronic renal failure. **b** Relationship between SPP at the proximal margin of the wound and probability of ischemic wound healing (modified from reference [19]). There is a significant relationship between SPP and wound healing, suggesting that assessment of the microcirculation is important. If SPP is 40–50 mmHg or more, the probability of wound healing is over 90%. Therefore, the SPP cutoff point of the diagnosis of CLI is considered to be 40–50 mmHg and the aim of revascularization is to achieve SPP of 40 mmHg or more

Assessment of limb ischemia is integral to diagnosing CLI. Traditional measurements that assess the macrocirculation, such as the ankle–brachial index (ABI) and ankle pressure, can be falsely elevated due to excessive calcification of the tibial artery, which is common in diabetic or ESRD-related CLI [14], and do not reflect blood flow below the ankle. Given the limited utility of assessment of the macrocirculation (Fig. 2a), intense evaluation of the microcirculation with measures such as skin perfusion pressure (SPP) and transcutaneous oxygen pressure (TCPo2) is essential in the setting of CLI [15–21]. In particular, SPP is a more reliable tool for detecting severe PAD involving calcified vessels and predicting healing of ischemic wounds than other methods for evaluating the macrocirculation and microcirculation (ABI, ankle pressure, toe–brachial index, toe pressure, and TCPo2) [15, 18, 22]. Indeed, given its simplicity and being approved for clinical use, SPP is widely used in most Japanese vascular centers. According to Castronuovo et al. [19], SPP values of 40–50 mmHg or higher at the proximal margin of the wound are associated with a high likelihood (90% and more) of wound healing in critically ischemic limbs (Fig. 2b). Thus, an SPP value less than 40–50 mmHg is the cutoff for diagnosing CLI; it is a strong indication for revascularization. In the clinical setting, even if macrocirculation indicators such as ABI are abnormal, SPP, a measure of the microcirculation, can be preserved in some patients, and vice versa. Therefore, evaluation of the microcirculation should be routine in patients with rest pain or tissue loss. When SPP measurement is unavailable, TCPo2 is an alternative.

**Fig. 3:**
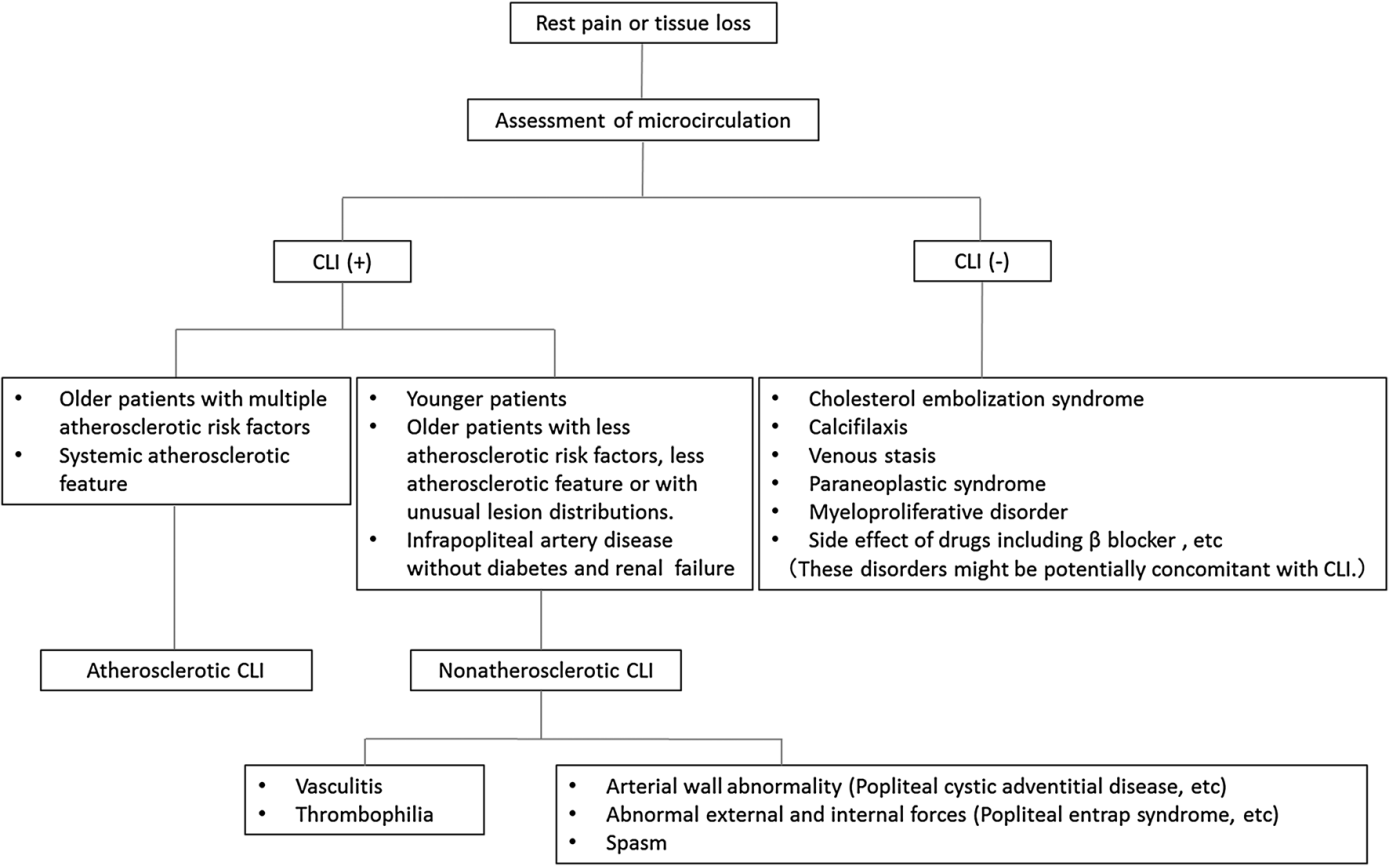
Diagnostic algorithm for atherosclerotic CLI, nonatherosclerotic CLI, and conditions other than CLI in patients with rest pain or tissue loss (modified from reference [28])

**Fig. 4:**
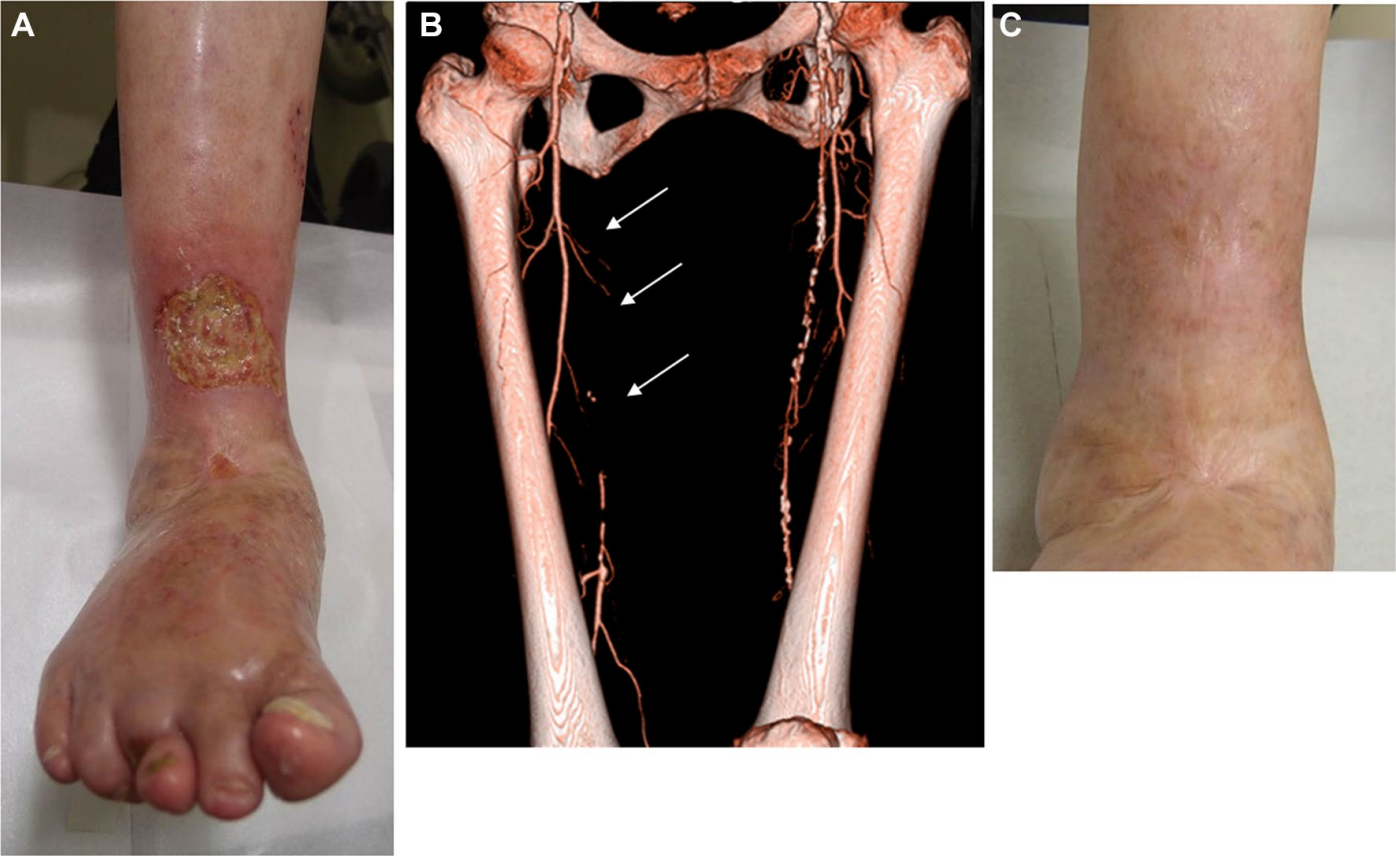
Venous stasis ulcer complicated by peripheral artery disease. **a** A 74-year-old female presenting with ulcer above the ankle. **b** Enhanced CT revealed long occlusion in the femoropopliteal artery (arrows). However, SPP around the ankle was almost 40 mmHg, suggesting preservation of microcirculation. **c** Wound management including compressive bandage facilitated complete wound healing 6 months later without revascularization

Atherosclerotic PAD is a major cause of CLI. Critically ischemic wounds consist of dry and wet wound. Typically, dry wounds can be observed in traditional CLI such as nondiabetic or non ESRD-related CLI. Caution needs to be taken to prevent sepsis when dry wounds might change to wet wounds because of the development of local bacterial infection after revascularization. Wet wounds can be frequently observed in diabetic or ESRD-related CLI, suggesting a significantly underlying bacterial infection (Fig. 1). If microcirculation in the foot is preserved, the cause of wound might be toe circulation disorder such as typical diabetic foot even though significant arterial lesions are evident. Nonatherosclerotic PAD (NAPAD) is potentially contaminated in our daily practice (Fig. 3). Underdiagnosis or misdiagnosis of NAPAD can lead to serious adverse outcomes that may be avoided or minimized with awareness of its distinctive symptoms and signs [23–28]. However, despite its common symptoms, NAPAD remains underappreciated compared to atherosclerotic PAD due to its low prevalence. Limb ischemia in younger patients warrants a high index of clinical suspicion for NAPAD. Even in older patients, most cases of NAPAD (e.g., infrapopliteal artery disease in patients without diabetes and renal failure) including vasculitis (e.g., scleroderma) and thrombophilia (e.g., antiphospholipid syndrome, essential thrombocytosis) might be misinterpreted as an atherosclerotic condition [29–31]. There is no vascular imaging feature in nonatherosclerotic CLI. Therefore, a high clinical index of suspicion for NAPAD with physical examination and laboratory tests is an integral part of the diagnostic evaluation. In cases where the microcirculation of the foot is preserved, other disorders including cholesterol embolization syndrome, calciphylaxis, venous stasis, systemic disorders (e.g., paraneoplastic syndrome and myeloproliferative disorder), and adverse effects of medications might be considered because patients with nonatherosclerotic CLI or conditions other than CLI might require different specific treatments (Fig. 4). Thus, correctly diagnosing the etiology of arterial lesions is essential for appropriate treatment strategy.

## Building a multidisciplinary team for infected neuroischemic wounds

**Fig. 5:**
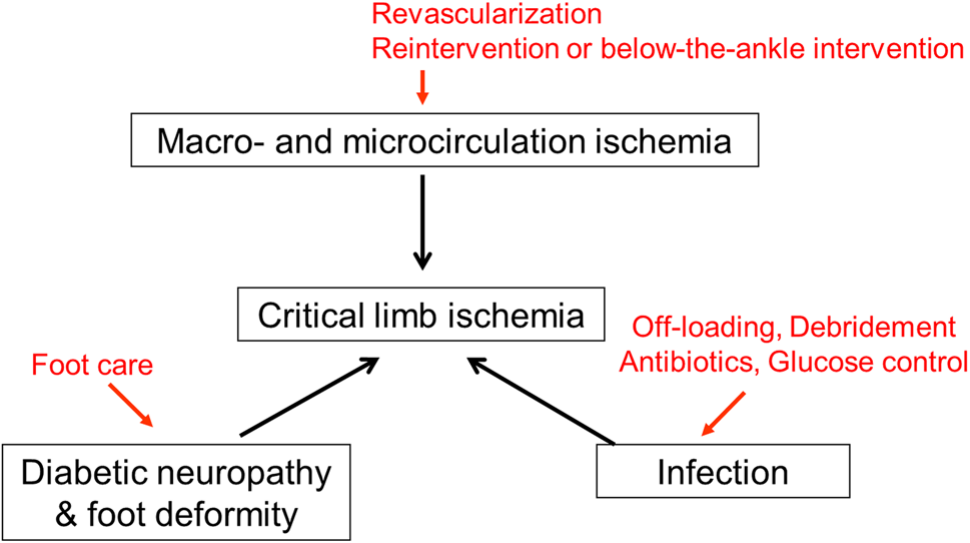
Complex pathology in critical limb ischemia. CLI can be characterized by multiple disorders. A comprehensive approach tailored to each patient including revascularization, infection control, and foot care is needed

Since the traditional categories of the Rutherford classification do not take into account coexistent bacterial infection in an ischemic wound, the WIFI classification has emerged in the context of wounds, ischemia, and infection [32]. More recently, with an awareness of the importance of concomitant foot deformity, the concept of arterial insufficiency, infection, and foot deformity (AID) concept has been proposed [33]. The extent to which ischemia, bacterial infection, neuropathy, and foot deformity are involved in each patient with CLI varies (Fig. 5).

**Fig. 6:**
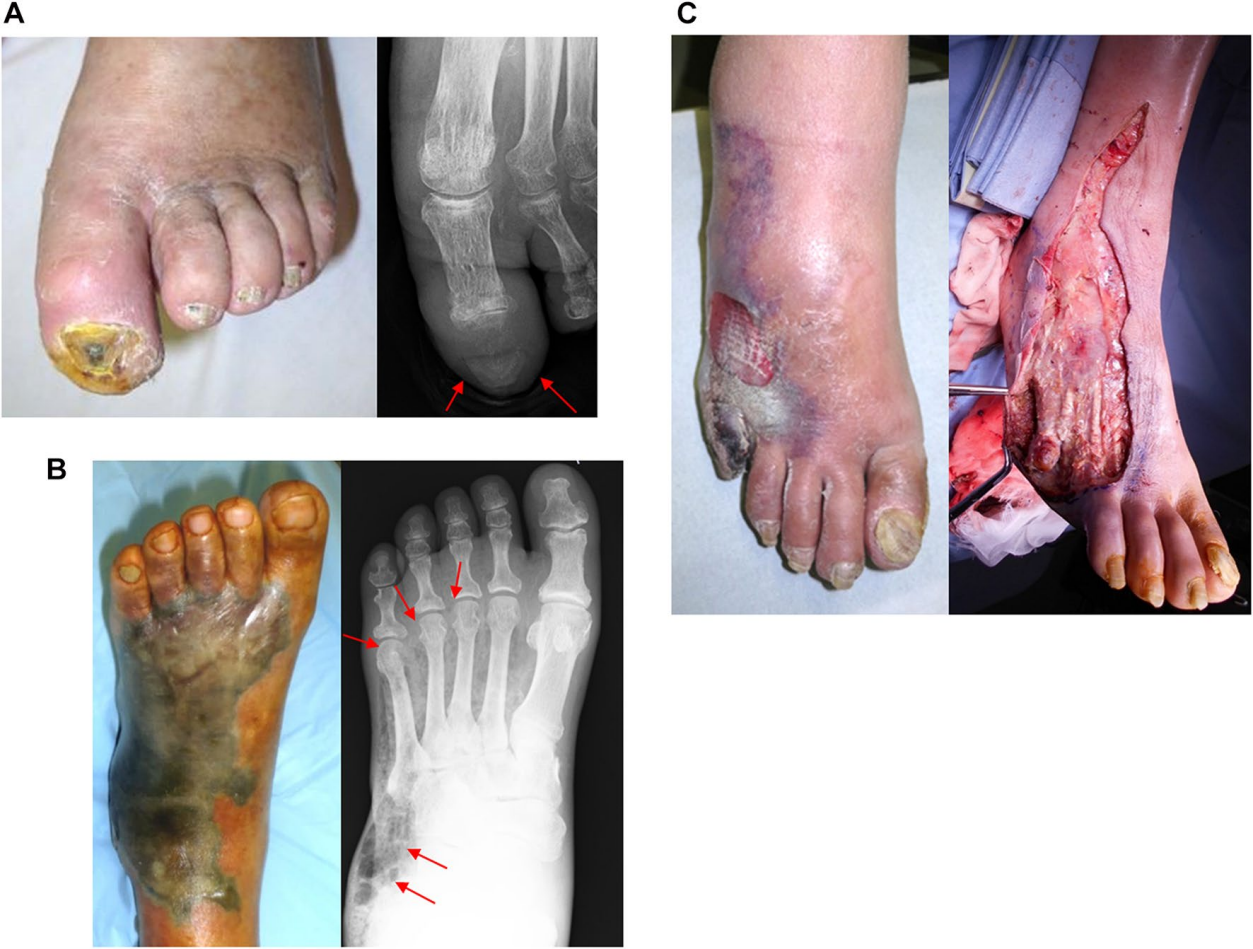
Potential for underlying severe infectious disease in patients with critical limb ischemia. **a** Osteomyelitis. Only a tiny necrotic lesion was visible on the top of the great toe. However, radiography demonstrated osteolysis of the distal phalanx, suggesting osteomyelitis. **b** Gas gangrene. Extensive gangrene on the dorsum of the foot was visible. Physical examination revealed snowball crepitus and radiography demonstrated the presence of air in the foot, suggesting gas gangrene. **c** Necrotizing fasciitis. Gangrene on the lateral side of foot with relative sparing of the skin was visible. Given the rapidly progressive course, severe inflammation, and high CRP value, necrotizing fasciitis was suspected. Emergent surgical incision demonstrated extensive necrosis of the subcutaneous fat and fascia with abscess formation

Although this is the time to liberally embrace contemporary endovascular procedures, standalone endovascular therapy alone cannot facilitate clinical success because many patients present with severe ischemic wounds, often with ongoing bacterial infection that can involve cellulitis, abscess, osteomyelitis, gas gangrene, or necrotizing fasciitis (Fig. 6). Therefore, in parallel with the assessment of foot ischemia and revascularization, assessment of the range and depth of bacterial infection is crucial. The bottom line is proactive wound management that encompasses timely liberal removal of devitalized structures (necrotic infected tissue), empirical or sensitivity-based antibiotic treatment, glucose control, off-loading, and epithelialization-stimulating dressings (Fig. 5) [34].

**Fig. 7:**
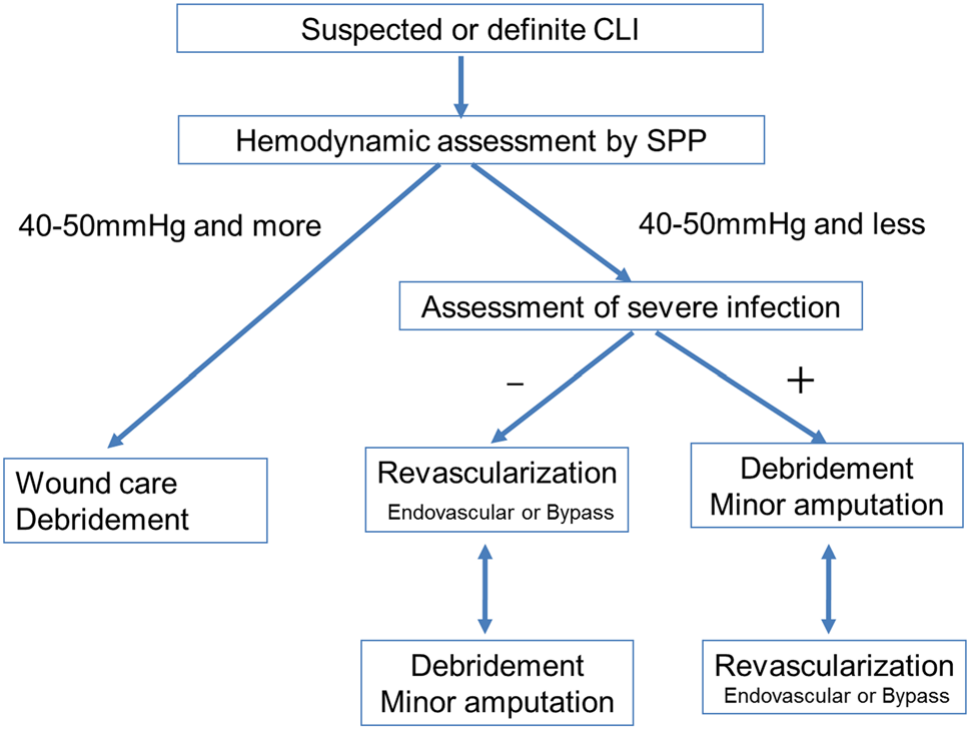
Comprehensive strategy for suspected or definite CLI. If the microcirculation is preserved (i.e., SPP is 40–50 mmHg and more), the foot is not affected by critical ischemia. Nonischemic reasons should be investigated. Wound care is the cornerstone of treatment. If the microcirculation is inadequate (i.e., SPP is 40–50 mmHg or less) and no severe bacterial infection is present, revascularization needs to be considered first. If the microcirculation is inadequate (i.e., SPP is 40–50 mmHg or less) and severe bacterial infection is present, debridement should be considered before revascularization

**Fig. 8:**
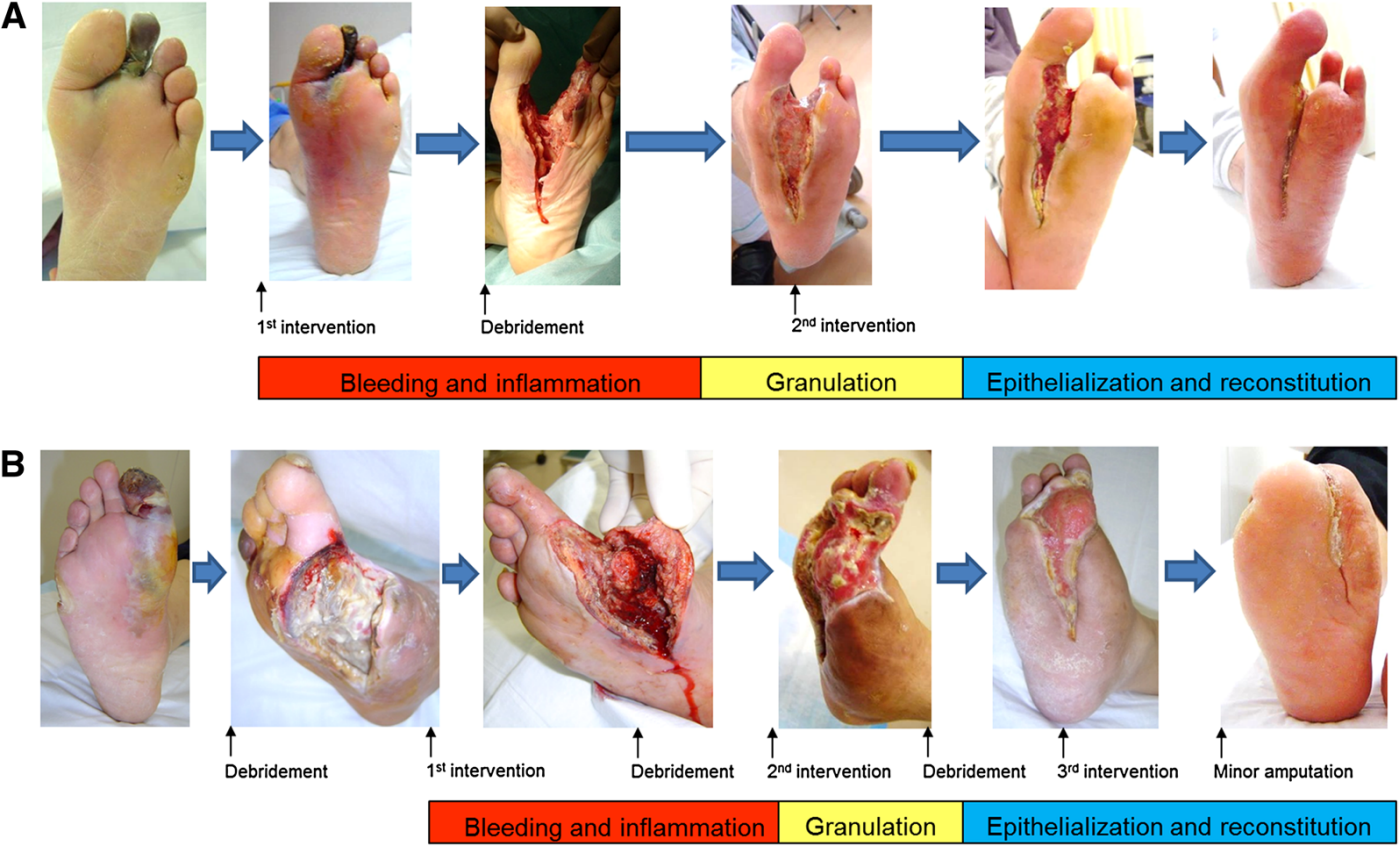
Practical collaboration of endovascular therapy and debridement/minor amputation. Caution needs to be undertaken about the development of bacterial infection after revascularization or preexisting severe bacterial infection before revascularization. The wound healing process after revascularization consists of 3 stages such as bleeding and inflammation stage, granulation stage, and epithelialization and reconstitution stage. **a** A case of development of bacterial infection after revascularization. A 65-year-old man with diabetes, ESRD, and second toe necrosis. Following the first intervention, severe cellulitis and abscess formation developed rapidly and extensively on the plantar aspect of the foot just below the gangrene on the second toe. Note the redness and swelling of the central foot just below the second toe gangrene. Urgent debridement and open drainage was implemented to prevent the further development of sepsis. Subsequently, granulation became evident after clinically driven reintervention. Finally, complete wound healing was achieved 6 months later. **b** A case of preexisting severe bacterial infection before revascularization. A 58-year-old man with diabetes, ESRD, and extensive infectious gangrene. Note the redness and swelling of the medial foot just below the first toe gangrene. Immediately after the first debridement of infectious tissue to prevent the development of sepsis, the first infrapopliteal intervention was performed. During the process of wound healing, clinically driven reintervention was required twice on the basis of skin perfusion pressure guidance, and a total of three times debridement or minor amputation was also executed. Finally, complete wound healing and gait acquisition were achieved 9 months later (modified from reference [4])

An essential process of wound healing includes: bleeding and inflammation, which are reactions to revascularization; and granulation, epithelialization, and reconstitution, which reflect cell proliferation. In cases where bacterial wound infection develops after revascularization, urgent debridement is required to prevent the development of sepsis and accelerate the wound healing process. In addition, in cases of preexisting severe bacterial wound infection (e.g., malodorous, high CRP), primary urgent debridement followed by endovascular revascularization needs to be considered. In particular, gas gangrene and necrotizing fasciitis might need urgent surgical intervention such as debridement or major amputation because these soft tissue bacterial infections can rapidly spread and become life threatening. In cases of severe inflammation with cellulitis, abscess, and osteomyelitis before or after revascularization, the priority for treatment can be placed on temporary debridement or minor amputation to prevent sepsis (Figs. 7, 8). In cases of uncontrolled infected wounds, major amputation or palliative care can be considered for patients who are not eligible for any intensive care and revascularization. In addition, rehabilitation to prevent disuse muscle atrophy is indispensable in CLI practice for functional capacity after wound healing and limb salvage. Therefore, since an individualized approach including specialists (plastic surgeons, orthopedic surgeons, etc.) and nurses can translate into improved management of CLI, a qualified team that organizes the disciplines of endovascular therapy and wound care should be established in each institution or regional medical network.

## Close follow-up for timely reintervention and debridement

Regular serial evaluation of the wound and microcirculation after revascularization until complete wound healing can facilitate early identification of wound worsening and timely clinically driven reintervention and debridement. Judging from the Japanese clinical experience, SPP evaluation or a similar technique on the day after the procedure, 2–3 days later, 7 days later, 1 month later, and every month thereafter might be a reasonable routine schedule [4]. In cases of unfavorable wound conditions (e.g., wound color, speed of granulation), temporary evaluation for severity of ischemia and infectious wound is recommended. Also, even if foot microcirculation is preserved, a couple of times of debridement or minor amputation might be needed to remove residual necrotic or infectious tissue and to control recurrence of infection in the wound until complete wound healing.

## Practical endovascular strategies

### Rationale for endovascular therapy

With the rapid evolution of endovascular techniques and technologies, the role of endovascular therapy for atherosclerotic CLI is expanding based on operators’ expectations of procedural success, risk of complications, patency rate, and potential of extensive reocclusion than before. Approximately, half of patients with CLI have multisegment disease with aortoiliac artery disease approximately in 20%, femoropopliteal artery disease approximately in 50%, and infrapopliteal artery disease in over 90% [20]. Aortoiliac intervention is durable, whereas femoropopliteal intervention is developing and infrapopliteal intervention remains underdeveloped [4, 35]. Ischemic tissue loss needs much more blood flow to heal than to resolve ischemic rest pain or to prevent recurrence of tissue loss. Since complete wound healing takes 3–6 months on average, endovascular therapy with liberal clinical-driven reintervention is acceptable on a clinical basis even if patency after endovascular intervention is short lived [9, 10, 36].

Reintervention is technically straightforward in most cases, and multiple reinterventions for recurrence of critical ischemia may be carried out as required until complete wound healing [4, 9]. Although the need for multiple reinterventions is common, when frequent repeat intervention is needed in a short period, shifting treatment toward surgical options can be considered if vascular surgeons proficient with bypass surgery are available and the patient can tolerate general anesthesia and bypass surgery with a good vein conduit. Also, endovascular therapy might be a last resort with symptomatic bypass occlusion in patients undergoing primary bypass surgery [37] and can serve as a bridge therapy to bypass surgery after the achievement of infection control.

Although there is a tremendous advancement of procedural success of endovascular therapy, some experts might consider hybrid therapy with endovascular therapy and bypass surgery for complex lesions. In cases of common femoral artery disease involvement, hybrid therapy consisting of endovascular therapy and endarterectomy can be considered although percutaneous common femoral angioplasty might be technically feasible and durable in the short term [38–41].

### Upstream revascularization

**Fig. 9:**
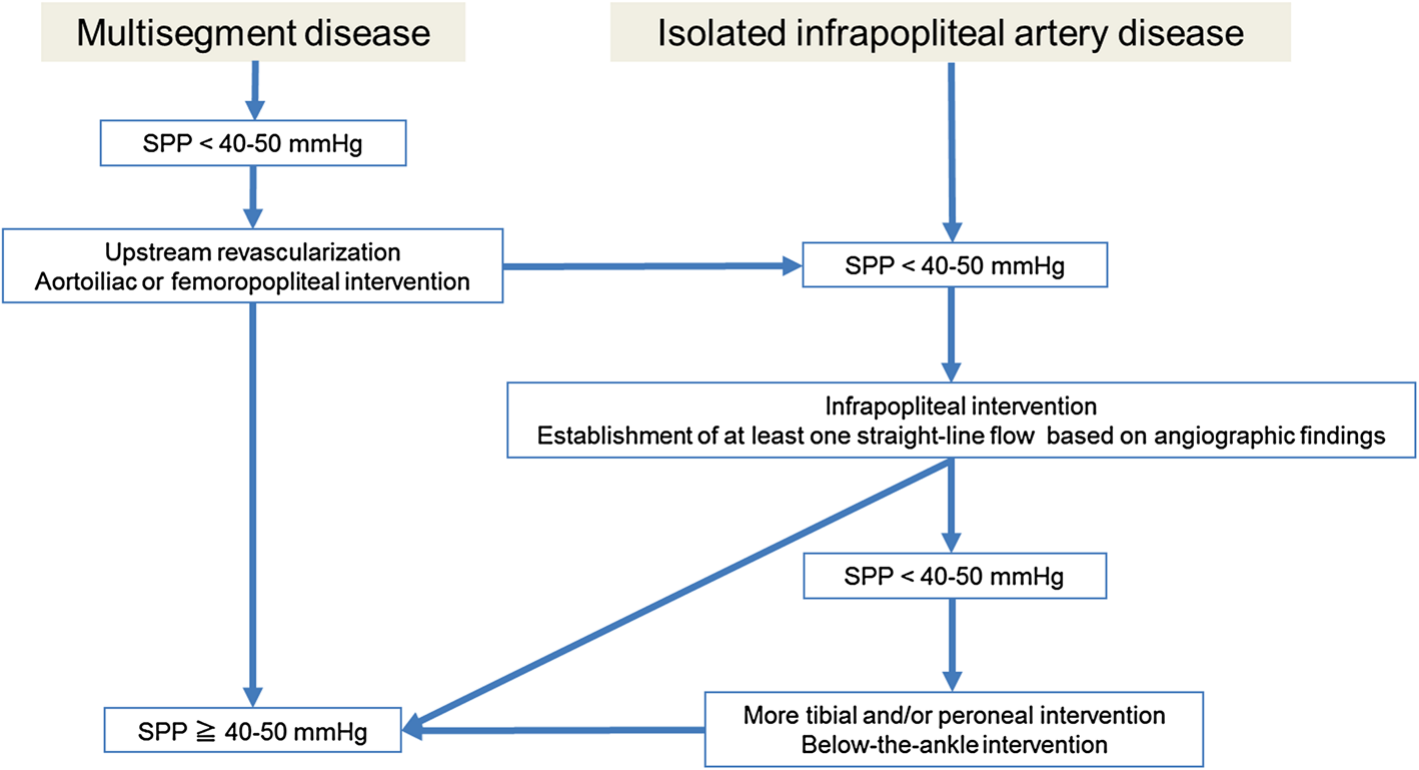
Endovascular strategy for multisegment disease or isolated infrapopliteal disease in CLI

**Fig. 10:**
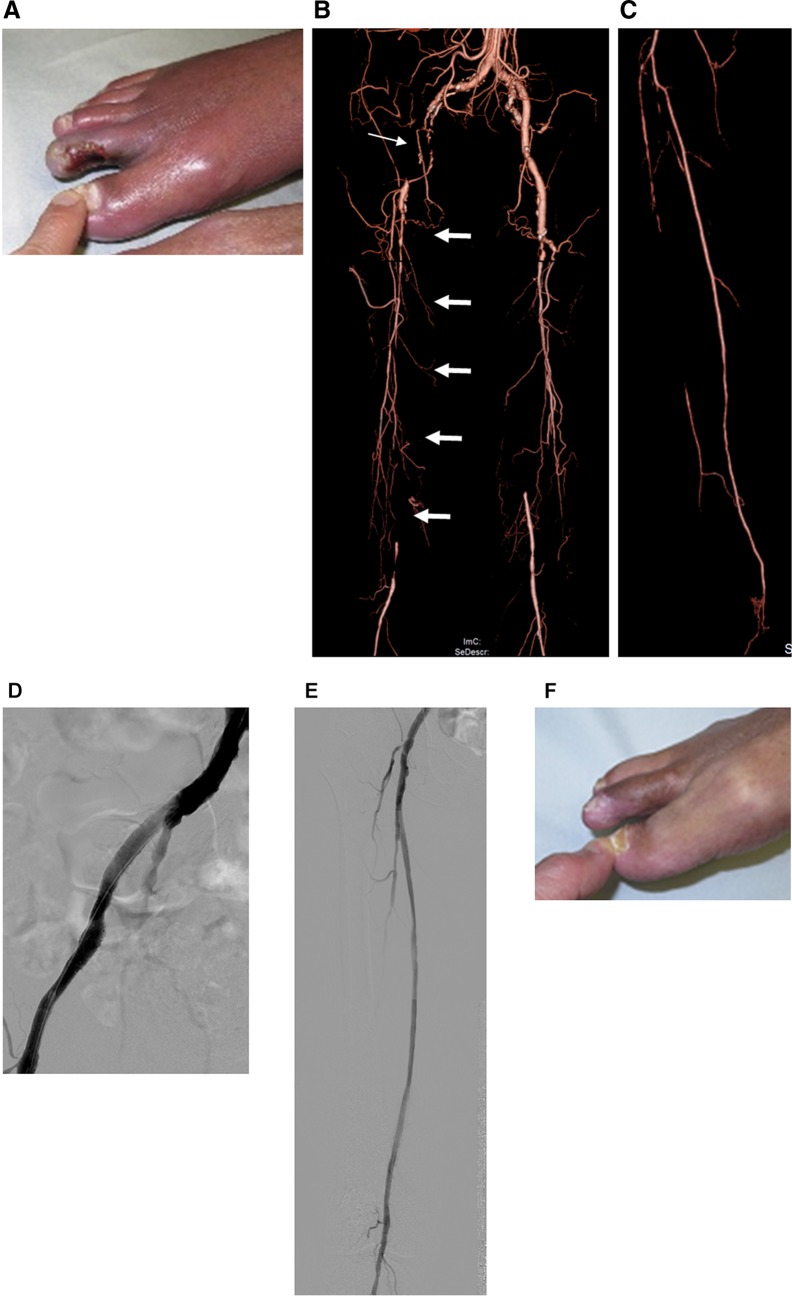
Representative case of staged procedures for multisegment disease presenting with tissue loss. **a** A 71-year-old male presenting with unhealing ulcer on the right second toe. Foot SPP was not measurable because of serious foot pain. **b** Enhanced CT revealed multisegment disease including occlusions in the right extrenal iliac (thin arrow) and femoropopliteal arteries (thick arrows). **c** Enhanced CT also revealed extensive occlusions in the right anterior tibial and peroneal arteries. **d** In the first session, right external iliac artery occlusion was successfully recanalized with stents. Given the concomitant femoropopliteal lesion complexity, contrast volume used, and procedure time, long occlusion in the femoropopliteal artery remained untreated in the session. Although rest pain significantly improved, the wound did not significantly heal with the foot SPP only 25 mmHg on the dorsum and 15 mmHg on the plantar, suggesting the need for further intervention. **e** In the second session, long occlusion in the right femoropopliteal artery was successfully recanalized with stents. However, given the potential of balloon-induced early reocclusion in the crural artery, the concomitant infrapopliteal artery remained untreated. **f** Foot SPP increased to 77 mmHg on the dorsum and 70 mmHg on the plantar, and the wound completely healed 5 months later

In patients with multisegment disease, the general rule is to increase upstream flow to the greatest extent possible because such revascularization might be enough to resolve rest pain and heal the wound in mild-to-moderate CLI even though downstream disease remains unrevascularized (Fig. 9) [4]. Revascularization of significant aortoiliac artery disease should be prioritized because aortoiliac intervention is an established practice, given its durability and excellent patency (Fig. 10). As for concomitant complex femoropopliteal disease, if aortoiliac revascularization is implemented, whether to do femoropopliteal revascularization depends on how clinical symptoms or signs can improve or how microcirculation of the foot can improve after aortoiliac revascularization (Fig. 10). If the concomitant femoropopliteal lesion is simple, femoropopliteal intervention might be implemented in the single setting with aortoiliac intervention. Femoropopliteal artery disease without aortoiliac artery disease should be revascularized if there is insufficient microcirculation in the foot.

### Establishment of one straight-line flow with infrapopliteal intervention

**Fig. 11:**
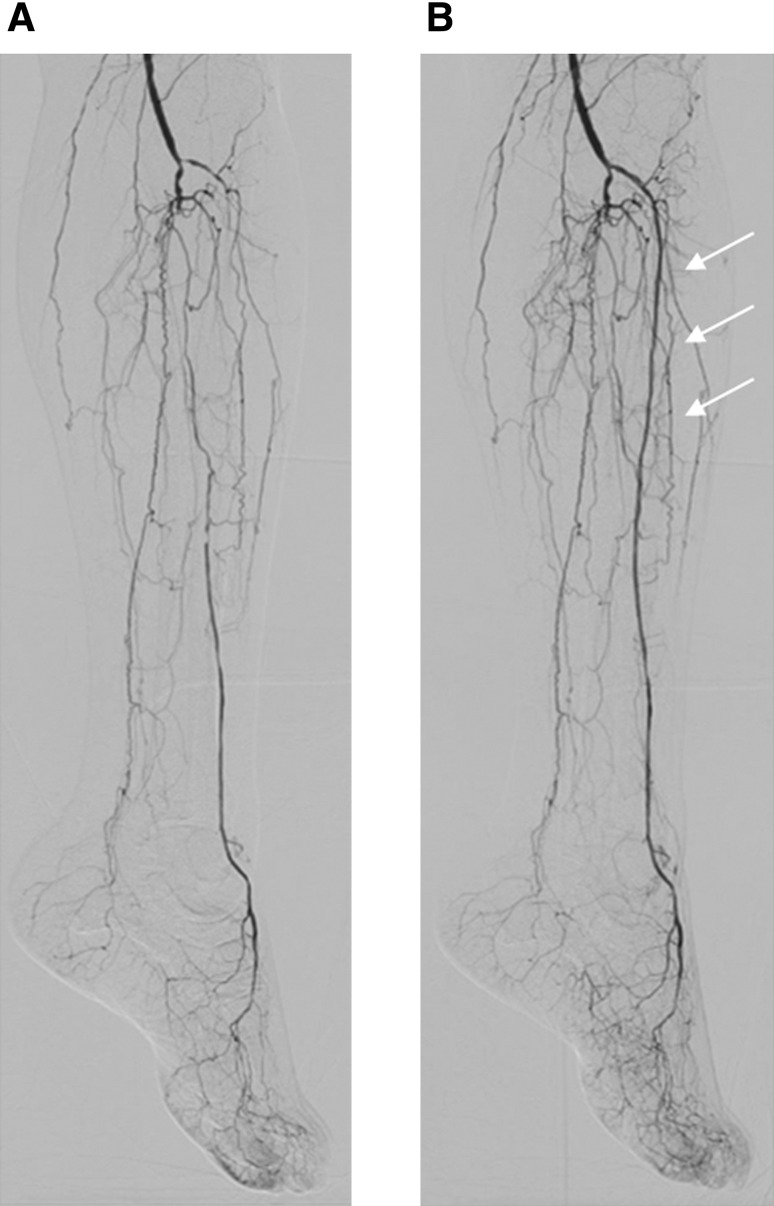
One straight-line strategy. **a** Baseline angiography. **b** Final angiography. In the setting of multivessel occlusion, the anterior tibial artery was successfully recanalized based on the interpretation of angiographic findings, suggesting the establishment of one straight-line flow to the foot (cited from reference [9])

In cases of isolated symptomatic infrapopliteal artery disease, or if no improvement of rest pain and tissue loss (ulcers and gangrene) persists with insufficient microcirculation of the foot following waiting to see the clinical effect of upstream revascularization, infrapopliteal intervention can be considered (Fig. 9). Since the hallmark of symptomatic infrapopliteal artery disease is multivessel occlusion, the goal of infrapopliteal intervention should be establishing at least one straight-line flow to each foot (Fig. 11). With advances in device technology and proliferative endovascular techniques, current procedural success rates could reach approximately 90% [42–45]. Based on the interpretation of angiography findings, an occluded vessel that seems technically feasible to reconstitute should be the primary target of endovascular intervention, in terms of the safety and effectiveness trade-off. The use of long balloons for tandem lesions including healthy-looking segments should be avoided because of the potential risk of balloon injury-induced reocclusion in originally nonoccluded segments. If no clinical improvement is observed without sufficient improvement in microcirculation after the first intervention, further intervention such as tibial/peroneal intervention or below-the-ankle intervention should be considered (Fig. 9). Based on the Japanese experience with SPP, microcirculation might improve gradually by a couple of days to 1 week after intervention.

### As many as possible strategy

**Fig. 12:**
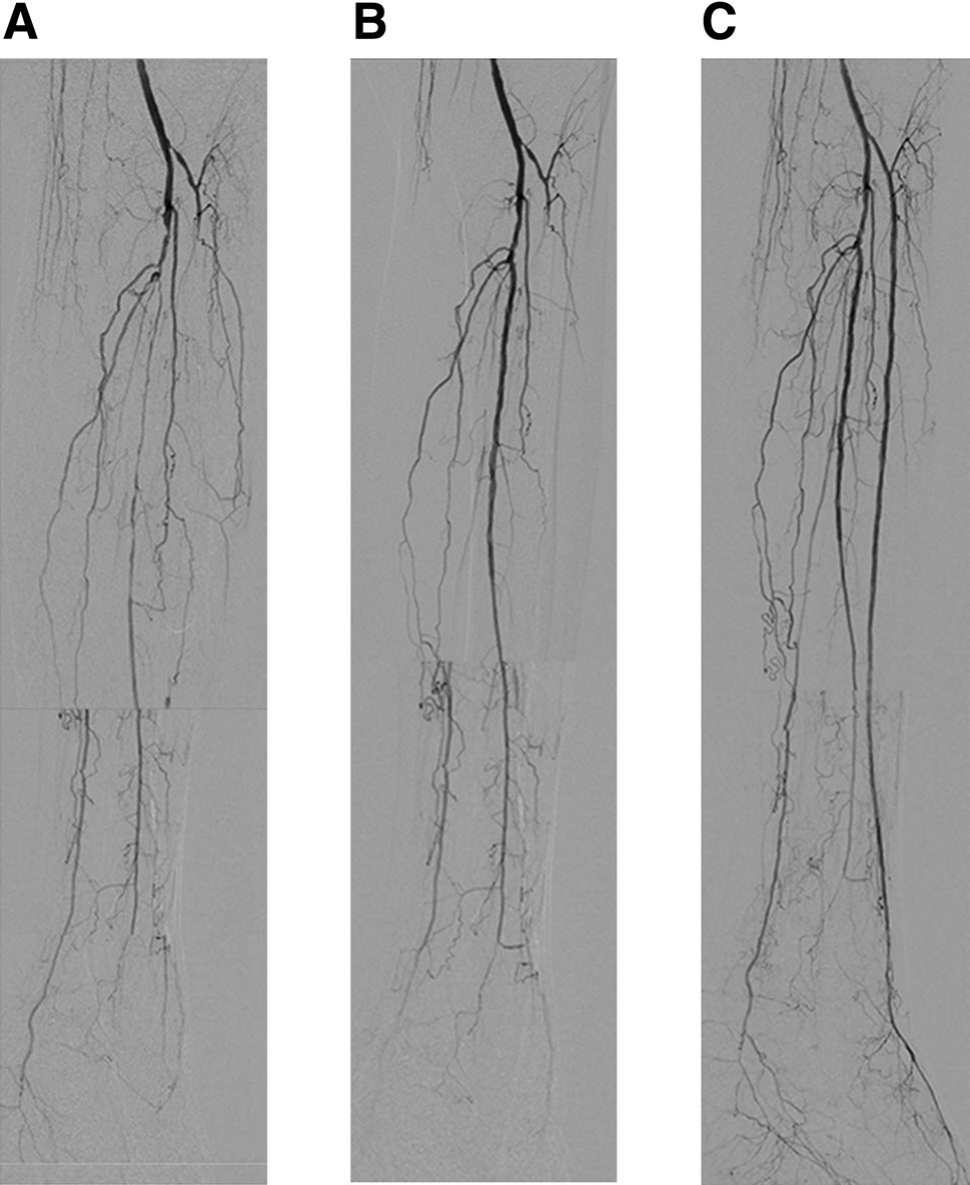
As many as possible strategy. **a** Baseline angiography. Note triple vessel disease in the infrapopliteal artery. **b** Post first intervention. During the first intervention, multiple stenotic areas in the peroneal artery were treated because it was technically straightforward. **c** Post second intervention. Since foot microcirculation after the first intervention was insufficient for wound healing (SPP 15 → 30 mmHg on the dorsum, 13 → 15 mmHg on the plantar), a second intervention was attempted. Since the reconstituted vessel in the distal anterior tibial artery became clear, recanalization of the long occlusion in the anterior tibial artery was successful. SPP increased to 52 mmHg on the dorsum and 42 mmHg on the plantar, and the wound healed completely

Opening as many tibial or peroneal arteries as possible can certainly provide robust blood flow to a severely ischemic foot and may enhance wound healing [46]. Therefore, given that over 50% of patients require clinically driven reintervention within 6 months because of a high rate of restenosis [9], this strategy is practical if technically feasible. Endovascular recanalization can be considered for simple to complex lesions. This strategy can be implemented in the first session or in subsequent sessions while waiting to see the clinical effect of the first session (Fig. 12).

### Controversy over the angiosome

**Fig. 13:**
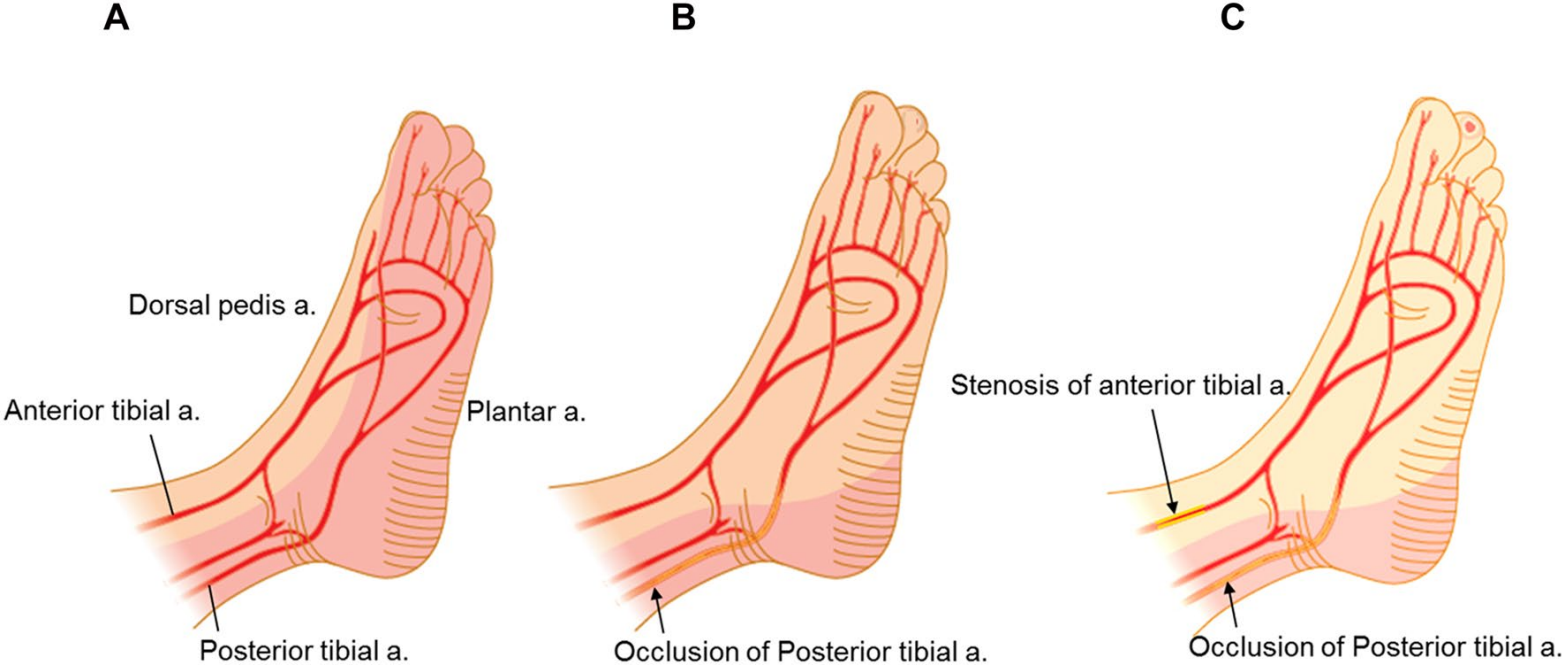
Schematic representation of foot perfusion in theory and the practical clinical perfusion territory of a single-source artery (modified from reference [4]). **a** Perfusion in the normal subject. **b** Clinical perfusion by a single-source artery. In cases of posterior tibial artery occlusion, the practical territory of the anterior tibial artery extends to encompass the normal territory of the adjacent source artery (posterior tibial artery) through the pedal arch, branches, and arterial connections. **c** A non-healing ulcer on the second toe (plantar side) in the setting of subsequent stenosis in the anterior tibial artery. Endovascular revascularization can be expected to significantly increase overall blood flow to the foot and the wound

There is a debate over the utility of angiosome-oriented intervention. To begin with, the angiosome might be misunderstood. The original concept of the angiosome, introduced by Taylor and Palmer in 1987, was in the context of flaps for skin healing. It was described as a 3-dimensional volume of skin, soft tissue, and bone supplied by a single-source artery and its branches that cannot be assessed in the setting of occlusion of adjacent arteries [47]. Given that symptomatic infrapopliteal artery disease can be characterized by multivessel occlusion, the use of the angiosome concept is not appropriate in the field of infrapopliteal revascularization. However, a 2-dimensional angiosome as a uniform map of vascular territories with clear boundaries has emerged in the field of surgical and endovascular treatment for CLI approximately 10 years ago [48–50]. Despite the lack of randomized comparative studies, there has been an increase in attempts to revascularize the artery feeding the 2-dimensional angiosome where ulcers or gangrene exists. On the other hand, more recent studies have raised objections to this approach that seems appropriate in theory [4, 9, 51–53]. Differences in benefits of the 2-dimensional angiosome theory in published studies could be due to the multifactorial nature of CLI. Of great interest, the most recent study using SPP found no significant difference in microcirculation between direct and indirect revascularization, and that approximately half of the revascularized feet had a change in microcirculation that was not consistent with the 2-dimensional angiosome theory [54]. In addition, a unique approach using indigo carmine angiography visually demonstrated that the area of foot perfusion is not consistent with the extent of crural artery occlusions [55]. Furthermore, a European group reported similar findings using laser Doppler flowmetry and tissue spectrometry [56]. The main reasons for these findings might be related to a unique 3-dimensional arterial network in each extremity with CLI (Fig. 13). Therefore, the primary target vessel should be the vessel where recanalization seems technically feasible based on the interpretation of angiographic findings and the operator’s skill.

### When to do below-the-ankle intervention

**Fig. 14:**
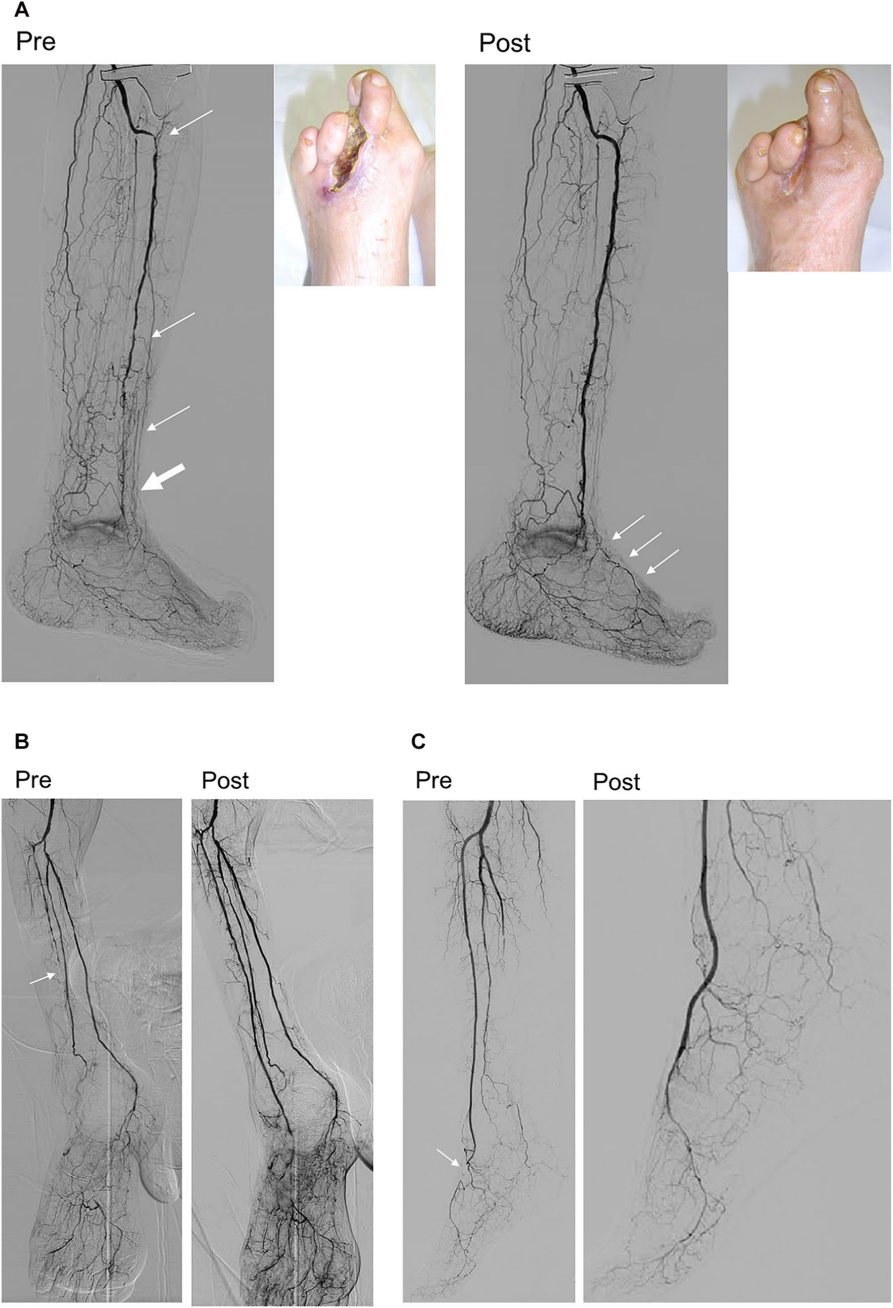
Types of below-the-ankle lesions. **a** Separate lesion type. At baseline, the anterior tibial artery lesions (thin arrows) were distinct from the below-the-ankle lesion, which involved an occluded dorsalis pedis artery through the reconstituted segment just above the ankle (thick arrow). Only above-the-ankle intervention for the anterior tibial artery was attempted to establish straight-line blood flow to the foot (cited from reference [61]). Although the occluded dorsalis pedis artery was left untreated, foot skin perfusion pressure increased from 25/32 to 37/64 mmHg (dorsal/plantar), leading to complete wound healing with debridement. **b** Serial lesion type. Baseline angiography showing extensive serial lesions from the anterior tibial artery to the dorsalis pedis artery beyond the ankle joint (arrow). Below-the-ankle intervention for the occluded dorsalis pedis artery was performed in the single setting with above-the-ankle intervention for the occluded anterior tibial artery to establish straight-line blood flow to the foot. **c** Isolated lesion type. Baseline angiography showed short segment occlusion in the dorsalis pedis artery (arrow) with diffuse involvement of the peroneal artery and a long occlusion in the posterior tibial to plantar arteries. Below-the-ankle intervention for the occluded dorsalis pedis artery was performed to establish straight-line blood flow to the foot (cited from reference [57])

**Fig. 15:**
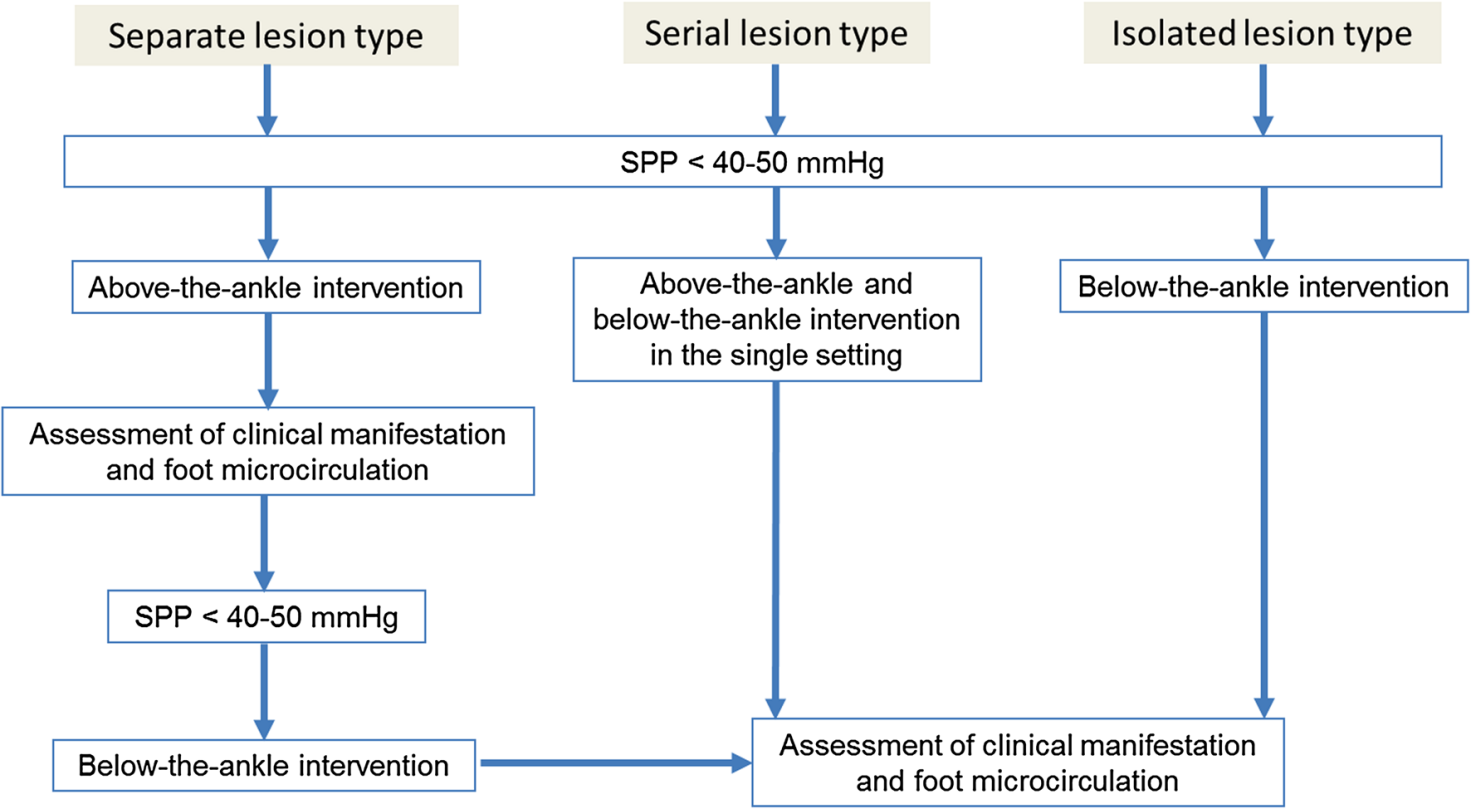
Endovascular strategy for below-the-ankle lesions. In cases where the below-the-ankle lesions are separate from the above-the-ankle lesions (separate lesion type), below-the-ankle intervention is not recommended during the first intervention because complications related to endovascular procedure and post-intervention reocclusion can potentially be catastrophic. In cases of extensive serial lesions beyond the ankle joint (serial lesion type) or isolated below-the-ankle lesions (isolated lesion type), treating the below-the-ankle lesions is recommended in the first intervention to improve foot microcirculation

Since the preliminary reports were initially published approximately 10 years ago from Asia [57, 58], there has been increasing awareness of the importance of below-the-ankle intervention for some patients [59, 60]. Given that the severity of pedal arch is significantly associated with wound healing, below-the-ankle intervention based on an assessment of the microcirculation is the final frontier [9]. However, indications for below-the-ankle intervention based on the 3 angiographic types (serial, isolated, and separate) should be emphasized; In cases of below-the-ankle lesions that do not connect to above-the-ankle lesions (separate lesion type), we recommend leaving the below-the-ankle lesions alone because below-the-ankle lesions will likely be affected by restenosis and reocclusion than can be potentially catastrophic or disastrous. If significant improvement in the microcirculation of the foot is achieved only after above-the-ankle intervention, the wound might completely heal even though below-the-ankle lesion would remain unrevascularized (Fig. 14a) [61]. Below-the-ankle intervention should only be considered if no clinical improvement with sufficient microcirculation is observed after above-the-ankle intervention. In cases of extensive occlusion beyond the ankle joint (serial lesion type), below-the-ankle lesions might be treated after above-the-ankle intervention in the single setting (Fig. 14b). Isolated below-the-ankle lesions (isolated lesion type) with one or more preexisting straight-line flows above the ankle is also the indication of below-the-ankle intervention (Fig. 14c). An endovascular algorithm for below-the-ankle lesions is shown in Fig. 15.

## Awareness of anatomical variants for endovascular therapy

Increasing awareness of lower limb artery anatomical variations can improve patient care. Although a persistent sciatic artery is a well-recognized anatomical variant in the suprapopliteal artery segment, variants in the popliteal artery branching pattern consisting of aplasty or hypoplasty of the tibial artery, a high takeoff for the tibioperoneal artery, trifurcation, and anterior tibioperoneal trunk are also not uncommon [62]. Given that approximately 10% of infrapopliteal arteries have variants, with the type 3A variant characterized by an aplastic posterior tibial artery and a hypertrophied peroneal artery connected to the plantar artery being the most common, differentiating occlusion from anatomical variation is a challenging task in severe infrapopliteal artery disease. When an infrapopliteal variant is observed in one extremity, there is a 28–50% probability of the same pattern on the other side. Keeping in mind the possibility of underlying infrapopliteal variations is the key to their successful identification and outcomes [62–64].

## Conclusions

Amid a pandemic of PAD with diabetes and ESRD in Asia, endovascular therapy represents a paradigm shift in the treatment of CLI. However, from a clinical perspective, a comprehensive patient-oriented approach, rather than a one-size-fits-all approach, is the key to clinical success. Therefore, harmonization of revascularization and wound management into a coordinated multidisciplinary approach that goes beyond a specialty-based framework is vital for the treatment of CLI.
